# Management of Antiphospholipid Syndrome

**DOI:** 10.3390/biomedicines8110508

**Published:** 2020-11-17

**Authors:** Amine Ghembaza, David Saadoun

**Affiliations:** 1 Groupe Hospitalier Pitié-Salpêtrière, Département de Médecine Interne et Immunologie Clinique, Sorbonne Universités AP-HP, F-75013 Paris, France; amine.ghembaza@aphp.fr; 2 Centre National de Références Maladies Autoimmunes Systémiques Rares et Centre National de Références Maladies Autoinflammatoires et Amylose Inflammatoire, Sorbonne Universités, F-75013 Paris, France; 3 Groupe Hospitalier Pitié-Salpêtrière, Inflammation-Immunopathology-Biotherapy Department (DMU 3iD), Sorbonne Universités AP-HP, F-75013 Paris, France

**Keywords:** antiphospholipid syndrome, antiphospholipid antibodies, autoimmune disease, warfarin, direct oral anticoagulants

## Abstract

Antiphospholipid syndrome (APS), is an acquired autoimmune disorder characterised by thrombosis, pregnancy morbidity, and the presence of antiphospholipid antibodies (aPL). Although venous thromboembolism is the most common manifestation, thrombotic events in APS may also occur in virtually any vascular bed, with cerebral circulation being the arterial territory most commonly affected. As APS is a heterogeneous condition, its management should be tailored with a patient-centred approach based on individual risk assessment, which includes the aPL profile, concomitant auto-immune diseases, and traditional cardiovascular risk factors. Although literature data are conflicting regarding primary prophylaxis, there is some evidence indicating that antiplatelet agents may reduce the risk of a first thrombotic event in individuals with a high-risk profile. In patients with thrombotic APS, current evidence-based guidelines recommend lifelong vitamin K antagonists (VKAs), preferably warfarin. The optimal intensity of anticoagulation following arterial thrombosis remains controversial. Arterial thrombosis should be treated either with high-intensity warfarin at a target INR > 3.0, or low-dose aspirin (LDA) combined with moderate-intensity warfarin (INR 2.0–3.0). It is recommended to avoid direct oral anticoagulants (DOACs) in patients with high-risk APS, mainly those with triple-positive PL and previous arterial events. They would only be used exceptionally in selected patients with low-risk venous thromboembolism (VTE). In low-risk VTE patients currently treated with a DOAC due to warfarin intolerance or a previous unstable International Normalized Ratio on warfarin, the decision of continuing DOACs would be taken in carefully selected patients. In women with obstetric APS, the combination therapy with LDA plus heparin remains the conventional strategy.

## 1. Introduction

Antiphospholipid syndrome (APS) is an acquired autoimmune disorder first described in 1983, characterised by thrombosis (in the venous, arterial, or microvascular circulation) and pregnancy morbidity, in the setting of persistently positive antiphospholipid antibodies (aPL): lupus anticoagulant (LA), anti-beta 2 glycoprotein 1 (anti-ß2GP1), and anticardiolipin antibodies (aCL). Persistent aPL positivity is defined as two or more consecutive readings at least 12 weeks apart [1,2]. The prevalence of APS in the general population is estimated to be 40–50 per 100,000. In autoimmune disease, particularly systemic lupus erythematosis (SLE), aPL can be detected in up to 40% of patients, but only one third of them will eventually develop clinical manifestations [3,4]. There are two main clinical patterns of APS, thrombotic and obstetric APS. Thrombotic APS most commonly presents with venous thromboembolism but also may present with arterial thrombosis, mainly stroke, with a prevalence of 53%, and 20%, respectively [5]. Obstetric APS is the most frequent treatable autoimmune disease during pregnancy [6]. It refers to patients who meet laboratory criteria for APS and have experienced prior pregnancy complications, including miscarriage, unexplained foetal death, and premature birth, with recurrent early miscarriage being the most common clinical scenario [7]. In contrast to thrombotic APS, obstetric events related to aPL are mainly due to complement activation and proinflammatory effects of aPL on decidual cells and trophoblasts without a real thrombophilic state, which result in defective placentation [8]. However, why some women with obstetric APS develop thrombosis after pregnancy, while others do not, still remains unanswered. In a retrospective study, thrombosis events after pregnancy morbidity related to APS occurred in 63.5% of cases. Those women were more likely to be younger, had more cardiovascular risk factors, superficial venous thrombosis, and heart valve disease than patients with only obstetric APS [9]. The adjusted Global Antiphospholipid Syndrome Score (aGAPSS) is a validated tool, including both aPL and conventional cardiovascular risk factors, predicting the likelihood of developing new thrombosis in an APS patient [10]. In some cases, both thrombotic and obstetric APS may coexist in the same woman (the so-called full-blown APS) [11].

APS may occur in isolation, and thus, it is said primary APS, or coexist with another autoimmune disease, mainly SLE. Other conditions might be associated with positive aPL, such as infections, malignancy, and drugs. In these cases, the antibodies are usually transient, at low titre, and without clear association with clinical manifestations of APS [12]. The primary APS affects predominantly younger adults of both genders with a median age of 40 years [13]. On the other hand, a marked female predominance has been documented in secondary APS, mainly SLE-associated APS [14]. In this review, we will summarize the current best evidence on the management of patients with APS, with a special focus on the use of direct oral anticoagulants.

## 2. Antiphospholipid Antibodies

Antiphospholipid antibodies (aPL) are a heterogeneous family of autoantibodies directed against phospholipids and/or phospholipid binding proteins, mainly, LA, anti-ß2GP1, and aCL [1]. Besides APS, these auto-antibodies are seen in other circumstances, such as infections, medications, and malignancy [3]. LA appears to be the strongest predictor of thrombotic risk for both venous and arterial thrombosis with a 5- to 16-fold increase in the odds ratio of thrombosis than the control, followed by anti-b2GPI, correlating mainly with arterial rather than venous thrombosis. Conversely, the aCLs are thought to carry the weakest risk of thrombosis, which correlate mainly with venous thrombosis [15]. In a prospective Italian study, hypertension and the presence of lupus anticoagulant were identified as independent risk factors for the development of thrombosis in asymptomatic individuals with aPL [16].

Selected screening for aPL in the setting of venous and/or arterial thrombosis may help to identify those patients who are more likely to have APS. Table 1 depicts the main indications for aPL testing. According to the British Society of Hematology in its addendum, patients with newly diagnosed unprovoked venous thromboembolism should be tested for Immunoglobulin G (IgG) and Immunoglobulin M (IgM) anti-ß2GP1 and aCL at the time of diagnosis. Patients who react positively to one or two solid-phase antibodies at presentation should be tested for those antibodies and for LA at three months [17]. To avoid false-positive LA with vitamin K antagonists (VKAs), patients should be tested after switching to low-molecular-weight heparin (LMWH) for one to two weeks, and then a blood sample should be collected 12 h after the last subcutaneous injection of LMWH [17].

Previous data indicate that all APS patients do not carry the same risk of thrombosis. In fact, ‘triple positivity’ denotes the presence of all three antibodies (i.e., presence of LA, aß2GP1, and aCL) and those patients carry the greatest risk of recurrent thrombosis. Furthermore, triple positivity was associated with a fourfold increased risk of recurrent thrombosis than single or dual positivity [17]. Furthermore, IgG anticardiolipin antibodies are typically considered the most relevant isotype compared with IgM [18].

Many non-criteria aPL have been described so far, and the most promising to become clinically relevant are antidomain I β2GPI (anti-β2GPI DI) and anticomplex phosphatidylserine prothrombin (anti-PS/PT) [19]. Moreover, a new entity, seronegative APS, has been introduced since 2003. Patients with seronegative APS share many clinical features suggestive of classical APS with persistently positive non-criteria aPL, but not LA, aß2GP1, and aCL [20]. In a previous report, up to one third of seronegative APS patients were positive to at least one non-criteria aPL, including anti-PS/PT, anti-cardiolipin/vimentin antibodies (aCL/Vim), antiphosphatidylethanolamine antibodies (aPE), and antiphosphatidylserine (aPS) [21]. Another advantage of testing for anti-PS/PT is that they could detect nearly 57–86% of LA-positive patients. Moreover, unlike LA, anti-PS/PT is unaffected by anticoagulation treatment [21]. Although some guidelines recommend testing for aCL IgA, aß2GP1 IgA, aPS/PT IgG, and IgM in patients with a high suspicion of APS and who are negative for criteria antibodies, their incorporation into routine clinical practice has not been validated due to the lack of performance and standardization [22]. 

## 3. Diagnosis

Current classification criteria for the diagnosis of APS, according to the 2006 international consensus statement, require at least one clinical criterion (vascular thrombosis or pregnancy morbidity) and at least one laboratory criterion (presence of lupus anticoagulant, or positive test results for anticardiolipin antibodies or anti-b2GPI) [23]. It has been shown that confirmation of aPL positivity on two occasions at least 12 weeks apart was usually associated with persistent positivity over time, and is thus mandatory for a definite APS diagnosis [24]. These antibodies should test positive at medium-to-high titres (IgG/IgM anticardiolipin > 99th percentile or >40 GPL/MPL, and anti-b2 glycoprotein I > 99th percentile). It is worth noting that these diagnostic criteria have been developed for research purposes and their extrapolation in clinical practice should be validated. One of the biggest drawbacks of these criteria is that they do not include some, albeit less common, clinical and laboratory features, known as non-criteria manifestations. These include livedo reticularis, cardiac valvular disease, epilepsy, or thrombocytopaenia, among others [15].

Similar to thrombotic APS, some women fail to meet the current diagnostic criteria for obstetric APS as they have incomplete clinical or laboratory features according to the aforementioned criteria. These patients could be classified as non-criteria obstetric APS [6].

## 4. Catastrophic Antiphospholipid Syndrome

The term “catastrophic” antiphospholipid syndrome (CAPS) was introduced in 1992 to describe a life-threatening and fortunately infrequent subset of APS, which is characterized by rapidly progressive (days to several weeks) multiorgan failure and high associated mortality due to widespread small vessel thrombosis. CAPS is still associated with a significant mortality rate that might reach 30%; fortunately, it represents less than 1% of cases of APS [25].

The following four criteria are required for the diagnosis of definite CAPS: (1) involvement of three or more organs, systems, and/or tissues; (2) development of manifestations instantly or in less than 1 week; (3) histological confirmation of small-vessel occlusion in at least one organ or tissue; and (4) aPL positivity [26]. The pathophysiology of CAPS is not fully understood; however, it was speculated that various triggering factors, including but not limited to infections, surgery, trauma, and malignancy, may induce endothelial injury, which results in cytokines overproduction and thrombotic storm in the microcirculation [27]. CAPS may occur in de novo fashion or complicate an already known APS [28].

## 5. Management

Either in primary or secondary situation, the management of aPL-positive patients should include the following general measures: (1) a tight control of cardiovascular risk factors, to include high blood pressure, hypercholesterolemia, body weight, and avoidance of smoking; (2) patient-centred risk stratification and screening for patients with a high-risk aPL profile, which include aPL triple positivity, LA or persistently high aPL titres, history of thrombotic and/or obstetric APS, the presence of other auto-immunes diseases such as SLE, and the coexistence of traditional cardiovascular risk factors [29].

Many risk scores have been developed to assess the risk of developing thrombosis and pregnancy morbidity in patients with APS. While most of them are based mainly on aPL profile, the Global APS Score (GAPSS) includes aPL profile and other risk factors, namely arterial hypertension and hyperlipidaemia. Patients with a GAPSS score ≥ 10 might be considered to be at a higher risk of thrombotic events and therefore require closer monitoring, especially in high-risk prothrombotic situations, such as major surgeries, long flights, and immobilization [30].

**Figure 1:**
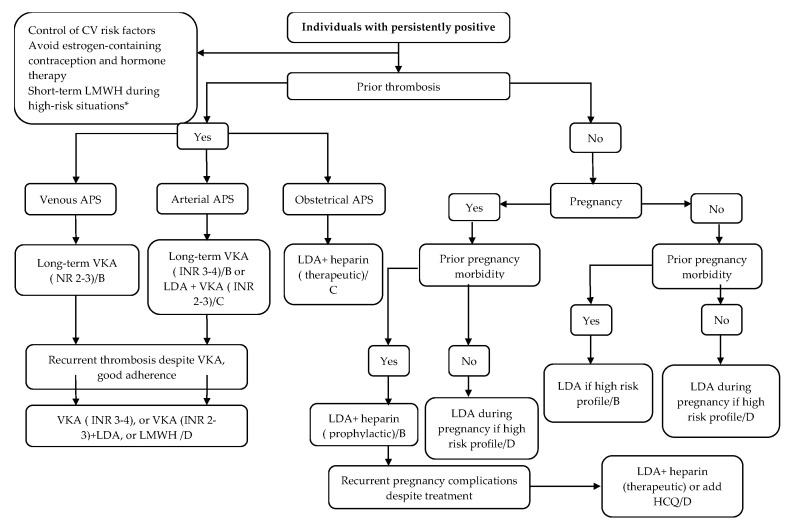
Treatment algorithm for antiphospholipid syndrome, adapted from [29]. aPL = antiphospholipid antibodies; CV = cardiovascular; DOAC = direct oral anticoagulant; INR = international normalized ratio; LDA = low dose aspirin; LMWH = low molecular weight heparin; VKA = vitamin k antagonist. Recommendation grade: B: consistent level 2 or 3 studies, or extrapolations from level 1 studies; C: level 4 studies or extrapolations from level 2 or 3 studies; D: level 5 evidence or troublingly inconsistent or inconclusive studies of any level. * Surgery, pregnancy/postpartum, and immobilization.

Figure 1 summarizes the treatment algorithm for antiphospholipid syndrome adapted from the European League Against Rheumatism (EULAR) guidelines [29].

### 5.1. Thrombotic APS

#### 5.1.1. Primary Prophylaxis

While the absolute annual risk of a first thrombotic event in positive aPL patients is less than 1% per year, this risk would increase to 5% when patients have additional thrombotic risk factors or other autoimmune diseases in addition to persistently positive moderate-to-high aPL [31]. Primary prophylaxis with aspirin in asymptomatic persistently aPL-positive carriers (without thrombotic or obstetric manifestations) was addressed firstly in a randomized controlled trial (RCT) that included 98 subjects (Antiphospholipid Antibody Acetylsalicylic Acid study, APLASA)**.** After a mean follow-up period of 2.2 years, no significant difference was found between aspirin (81 mg/day) and placebo in terms of the overall annual incidence rate of thrombosis [32]. Similar findings were observed in a prospective observational parallel study, including eligible patients who could not participate in APLASA [32]. However, this study should be interpreted with caution due to some limitations, such as the small sample size, short term follow-up, and inclusion of positive aPL patients with low titers or IgA isotype [32]. The role of low-dose aspirin (LDA) for primary prevention in asymptomatic aPL carriers was corroborated by a meta-analysis including 11, mainly observational, studies, with a more significant effect in patients with arterial thrombosis. Patients treated with LDA had a two-fold reduction of a first thrombotic event. However, subgroup analysis of only prospective and high-quality studies revealed no significant protective effect of LDA over no treatment [33]. A subsequent meta-analysis of five studies performed by the same group showed, after adjusting for cardiovascular risk factors, aPL profile, and treatment with hydroxychloroquine (HCQ), similar findings (HR: 0.43 [95% CI: 0.25–0.75]). As reported in the previous meta-analysis, primary prophylaxis with LDA reduced arterial but not venous events [34]. On the other hand, LDA-associated bleeding risk is a serious concern that must be taken into account. In fact, a meta-analysis including six RCTs showed that LDA was associated with an increased annual risk of major bleeding, with older age (>65 years), male sex, diabetes mellitus, and hypertension being the most significant associated risk factors [35]. In the setting of APS, a previous study of asymptomatic aPL patients with SLE demonstrated that the benefit of primary prophylaxis with LDA outweighed treatment-associated bleeding risk [36].

To assess the efficacy and safety of LDA in association with warfarin in primary prophylaxis, a randomized open-label controlled trial including 166 aPL-positive carriers showed no significant difference in terms of the number of thrombosis between patients treated with LDA (75 mg) alone versus LDA plus low-intensity warfarin (INR: 1.3–1.7) over a five-year follow-up period. More episodes of bleeding were found in the LDA plus warfarin arm [37].

Although not supported by high-quality studies, the task force at the 13th International Congress on Antiphospholipid Antibodies suggested long-term thromboprophylaxis with LDA at 75–100 mg/day in subjects with high-risk aPL profile, mainly individuals with triple positivity, LA positivity, isolated persistently positive aCL at medium to high titers, and those with other thrombotic risk factors. In patients with SLE who test positive for LA or aCL at medium-high titers, primary thromboprophylaxis with HCQ plus LDA at 75–100 mg/day should be considered [38].

More recently, EULAR recommends primary prophylaxis with LDA (75–100 mg/day) in asymptomatic aPL carriers in the following circumstances: (1) high-risk aPL profile with or without traditional risk factors, (2) patients with SLE and high-risk aPL profile, and (3) non-pregnant women with a history of obstetric APS only [29].

In summary, the decision of primary prophylaxis should be taken after a patient-centred risk stratification, which includes aPL profile (number, titer, isotype), coexistence of other auto-immune disease or an inherited thrombophilia, and cardiovascular risk factors. This assessment may identify patients who would benefit from primary prophylaxis. Some authors suggest transient thromboprophylaxis with LMWH during high-risk situations, such as surgery, pregnancy/postpartum, and immobilization [39].

#### 5.1.2. Secondary Prophylaxis

Undoubtedly, VKAs, mainly warfarin continued to be the treatment of choice in patients with thrombotic APS (treatment and secondary thromboprophylaxis). In case of venous thromboembolism (VTE), the intensity of treatment should be aimed at a target international normalized ratio (INR) of 2.0–3.0, for an indefinite period [38]. This treatment strategy showed an 80–90% risk reduction of recurrent venous thromboembolism [40].

Some observational studies have shown some benefits of high-intensity anticoagulant therapy (INR 3.1–4.5) over moderate-intensity warfarin in terms of recurrent thrombosis (INR 2.0–3.0) [41]. However, high-intensity warfarin did not further reduce the risk of recurrent thrombosis, on the basis of two subsequent RCTs [42,43]. Moreover, when combining these two RCTs in a meta-analysis, high-intensity warfarin was associated with a significant excess of minor bleeding compared to standard-intensity anticoagulation [43]. When a first provoked VTE event occurs in a patient with low-risk profile, then a limited duration of anticoagulation can be considered [29].

As opposed to venous thromboembolism, the optimal intensity of anticoagulation following arterial thrombosis remains controversial. In the Antiphospholipid Antibodies and Stroke Study (APASS), a prospective observational study, including 720 positive aPL patients with previous stroke event, warfarin with a target INR 1.4–2.8 was not proven to be superior to aspirin (325 mg/day) in stroke prevention over two years. The authors concluded that patients with previous stroke event and persistent aPL positivity not fulfilling classification criteria would be best treated as the general population, with LDA. Nevertheless, screening for aPL was performed only at the study entry, which raises the possibility of transient positive aPL [44].

To evaluate the efficacy of LDA in secondary thromboprophylaxis, particularly in arterial APS patients, an RCT including 20 APS patients (13 primary APS and 7 with SLE) with ischemic stroke showed that the combination of LDA (100 mg/day) and anticoagulation therapy (target INR 2–3) was superior to LDA alone, without a difference in terms of haemorrhagic complications between the two arms [45].

The task force at the 13th International Congress on Antiphospholipid Antibodies advised that patients with arterial thrombosis should be treated either with high-intensity warfarin at a target INR > 3.0, or low-dose aspirin (LDA) combined with moderate-intensity warfarin (INR 2.0–3.0). However, it should be taken into account that it was a non-graded recommendation [38]. EULAR guidelines were in line with these recommendations [29].

However, in a minority of APS patients, thrombotic events recur despite apparently adequate anticoagulation. These patients are deemed anticoagulant refractory [46]. This scenario is, in fact, quite infrequent and accounts for about 4.3% of all APS subjects [5]. Managing patients with anticoagulant-refractory thrombotic APS is a major challenge and based mainly on expert opinions. The common practice is to increase VKA anticoagulation intensity or to switch VKA to LMWH; subsequently, adjunctive treatment with HCQ, statins, complement inhibitors, or antiplatelet agent would be considered [46].

On the other hand, treatment with VKAs is often problematic, as they have a narrow therapeutic window, require regular monitoring of INR, multiple drug and dietary interactions, and the potential for variation of action in the presence of alcohol, intercurrent illness, exercise, and smoking [13].

Direct oral anticoagulants (DOACs) have quickly become attractive alternatives to VKAs. The main advantages of DOACs over VKA lie on their fixed-dosing protocol, more rapid and predictable anticoagulant response, no need for laboratory monitoring of coagulation tests, reduced major and intracranial bleeding, and fewer drug–drug and drug–food interactions [47]. Four direct oral active anticoagulants (DOACs), dabigatran, rivaroxaban, apixaban, and edoxaban, are approved in the general population for the prevention of stroke in patients with nonvalvular atrial fibrillation, deep venous thrombosis prophylaxis, treatment, and secondary thromboprophylaxis after an episode of venous thromboembolism in Europe and the United States of America [48].

In a meta-analysis of 47 studies including a total of 447 APS patients who received DOACs, 16% developed a recurrent thrombosis after a mean period of 12.5 months. The recurrent thrombosis occurred in 16.9% and 15% of patients receiving a factor Xa inhibitor (rivaroxaban or apixaban) or a factor IIa inhibitor (dabigatran), respectively. Compared to patients with single or dual positivity, those with triple positivity had a four-fold increased risk of recurrent thrombosis [49]. Besides aPL profile, patients treated for arterial thrombosis with rivaroxaban or apixaban had a twice higher risk of recurrent thrombosis compared to those with venous thrombosis. The authors concluded that APS patients with triple positivity and those with previous arterial manifestations are more prone to recurrent thrombosis when treated with DOACs [49].

In the RAPS trial, a non-inferiority randomized open-label study, 116 APS patients with at least one venous thromboembolism event or recurrence were assigned to receive either rivaroxaban 20 mg or to continue on warfarin. Although the primary endpoint, the mean percentage change of thrombin potential was not reached; the authors found no difference between the two groups in terms of rates of venous thromboembolism as a secondary outcome as well as no difference in adverse events, over a six-month follow-up period. Of note, triple aPL positivity was found in 12% and 20% of patients treated with rivaroxaban and warfarin, respectively. The authors concluded that rivaroxaban could be efficacious and safe in this subgroup of APS patients. However, this study was underpowered for clinical events [50].

The TRAPS trial, a non-inferiority randomized open-label trial comparing rivaroxaban with warfarin in triple-positive APS patients with a history of thrombosis, assessed the risk of thrombosis as the primary outcome. This study was stopped prematurely after the enrolment of 120 patients due to the occurrence of higher rate of thrombotic events (mostly arterial) in the rivaroxaban arm. Thrombo-embolic events and major bleeding occurred in 12% and 7% in the rivaroxaban group compared with 0% and 3% in the warfarin group, respectively. Noteworthy, four out of a total of seven recurrent thrombotic events in the rivaroxaban arm occurred in patients with previous arterial thrombosis [51].

A meta-analysis of the previous two RCTs (RAPS, TRAPS) did not find an increased risk of thrombosis in patients treated with rivaroxaban versus VKA at 6 months follow-up. It should be taken into account that 8 out of the 13 events in the TRAPS trial occurred beyond 6 months of follow-up [52]. In a more recent clinical trial, including 190 APS patients, the authors could not demonstrate noninferiority of rivaroxaban to dose-adjusted VKAs for secondary prevention of thrombosis [53]. In a systematic review of the literature including more than 400 patients, Dufrost et al. identified two main patient characteristics associated with recurrent thromboses in patients treated with DOACs: triple-positivity profile and history of arterial thrombosis [49].

The reasons for the excess of thrombotic events in APS patients treated with DOAC are not clear. Some authors hypothesised that this effect may be related to: (1) the single target of DOACs as compared to the effect of VKAs, which induce the inhibition of several coagulation factors; (2) inhibition of both extrinsic and intrinsic pathways of coagulation by VKAs, which results in the generation of less thrombin compared with DOACS; (3) lower compliance with DOACs in the absence of therapeutic monitoring; and (4) a requirement for a higher effective dose of DOAC to prevent arterial as compared to venous thrombosis. However, these explanations remain to be confirmed [51]. Table 2 summarizes the most relevant clinical trials comparing the efficacy and safety of DOACS versus VKAs in patients with thrombotic PS.

Based on the previous two RCTs (RAPS, TRAPS), the European Medicines Agency (EMA) recommended against the use of DOACs in APS patients, especially in those who are triple positive [54]. EULAR recommended against the use of rivaroxaban in APS patients with triple positivity [29]. The British Society for Haematology (BSH) recommends the use of VKAs for treatment and secondary prophylaxis in patients with venous thrombosis who are triple positive and those with arterial thrombosis. In the case of patients with non-triple-positive APS and venous thrombosis, BSH recommends warfarin therapy unless they are already on a DOAC and do not wish to switch to a VKA; continuation of the DOAC would be feasible after discussion with the patient. They advise to take into account the patient clinical history and experience with anticoagulants, which may favour patient adherence [17]. Moreover, DOACs may also be considered in exceptional cases in patients who have contraindications to VKA and those with difficulty of achieving and maintaining a target INR of 2.0–3.0 despite good compliance with VKA [54].

Whether or not anticoagulation should be withdrawn in APS patients after seroconversion is still a matter of debate. Seroconversion defines a subgroup of patients with a definite diagnosis of APS in whom aPL becomes persistently negative. In a systematic review including four retrospective studies, recurrences after anticoagulation discontinuation occurred in 0%, 0%, 10%, and 45.8%, respectively [55].

### 5.2. Obstetrical APS

Women with APS should benefit from pre-conception planning in order to inform the patient of potential maternal and foetal/neonatal risks associated with APS pregnancy [56]. In addition, in positive aPL women, oestrogen-containing oral contraceptive pills or oestrogen replacement therapy should be avoided due to their prothrombotic effects [57].

#### 5.2.1. Primary Prophylaxis

Regarding primary prophylaxis in pregnant women without prior morbidity, one RCT included 924 women with positive aPL who were randomly assigned to receive either aspirin 60 mg/day or placebo. Pregnancy outcomes did not differ significantly between the two groups [58]. In women (with or without SLE) with a high-risk aPL profile and without a personal history of thrombosis or pregnancy complications, EULAR recommends primary prophylaxis with LDA (75–100 mg/day) during pregnancy [29].

#### 5.2.2. Secondary Prophylaxis

Management of obstetric APS must include close monitoring performed by a multidisciplinary team during all of the pregnancy period and the postpartum.

LDA, LMWH, and unfractionated heparin (UFH) used as a single agent or in combination in obstetric APS have been examined by a plethora of studies. Without treatment interventions, only about 10–50% of pregnancies are reported to have been successful [59]. On the other hand, advances in treatment and management of obstetric APS yielded a live birth rate of 80% [60].

However, the optimal treatment strategies are still being debated today. In a meta-analysis, pregnant women, with previous recurrent spontaneous abortion, treated with LDA plus LMWH had significantly more live births compared with women who received LDA alone. Moreover, treatment with LDA alone did not show a protective action compared with placebo with respect to the live birth rate [61]. Similarly, a more recent meta-analysis including 12 RCTs confirmed the superiority of the combination of heparin (LMWH or UFH) and LDA in terms of both the live birth rate and birthweight. However, in patients without a history of thrombotic events, the association of LDA plus heparin was not superior to LDA alone in terms of the live birth rate [59].

Although the combination LDA plus heparin certainly provides an element of maternal thromboprophylaxis and may improve pregnancy outcomes, women with clinical and/or biological risk factors are at increased risk of adverse pregnancy outcomes in spite of that standard treatment. These risk factors include a personal history of thrombosis, SLE, foetal death, or early delivery for severe preeclampsia or placental insufficiency, and repeated LA or triple positivity [7]. Moreover, in PROMISSE, a prospective observational study of 724 patients, 44% of pregnancies in women with APS and positive LA resulted in adverse pregnancy outcomes despite treatment with heparin and LDA [62].

In women with pure obstetric APS (without prior thrombotic event), the current EULAR guidelines recommend treatment with LDA (75–100 mg/day) plus heparin at a prophylactic dose during pregnancy, regardless of the presence or the absence of a concomitant SLE [29]. LDA should be started before conception and stopped four weeks before the delivery, while heparin should be started as early as possible on confirmation of pregnancy, and continued for 3–6 weeks during the postpartum period, given the increased risk of thrombosis during this period [57]. Women who continue to have pregnancy complications despite this regimen and those with prior thrombotic complications (regardless of the pregnancy history) should be treated with LDA with heparin at a therapeutic dosage [29].

While the efficacy of this regimen with regard to maternal thromboprophylaxis is well established, low evidence still exists in terms of embryonic/foetal outcomes and second/third trimester complications [7]. In fact, refractory obstetric APS may occur in about 30% of cases, despite tight pregnancy monitoring and the use of treatment combination with LDA plus LMWH [57]. Some retrospective studies showed that the additional use of HCQ in APS women refractory to conventional therapy with heparin plus LDA was associated with significantly higher live birth [63,64]. However, the beneficial effect of HCQ in improving maternal and foetal outcomes in APS women should be investigated by well-designed RCTs. In fact, several clinical trials that assess the efficacy of HCQ administered as add-on to conventional therapy in women with obstetric APS are ongoing [65,66,67]. Some experts suggest adding low-dose corticosteroids (10 mg/day) during the first trimester of gestation to dual therapy with LDA plus LMWH in case of refractory obstetric APS [57]. A phase 2 clinical trial assessing the efficacy of certolizumab, a PEGylated tumour necrosis factor-alpha inhibitor, in women with a past history of pregnancy losses in spite of standard therapy with heparin and LDA is ongoing (NCT03152058).

### 5.3. Seronegative APS

The assessment and interpretation of non-criteria aPL should be performed by expert centres. These tests should be considered in patients with a high clinical suspicion of APS, such as those presenting with recurrent unexplained thrombosis, thrombosis at unusual sites, or women with recurrent pregnancy-related complications. This strategy may guide the type and the duration of anticoagulation [68]. 

As stated for patients with APS, the first step in the assessment of seronegative APS patients should focus on patient risk stratification, including aPL type and titers, traditional cardiovascular risk factors, and the clinical pattern. As no guidelines that address specifically the management of seronegative APS are available, treatment strategies could be extrapolated from data on patients with definite APS [68]. 

In a retrospective study that compared 65 women with seronegative APS with 83 seropositive APS and 31 healthy pregnant women, treatment with LDA and LMWH was significantly associated with live births in both seropositive and seronegative women compared with controls [69]. This finding was corroborated by a recent multicentre case series [70]. 

### 5.4. Other Therapies

#### 5.4.1. Hydroxychloroquine

Besides its immuno-modulatory activity, HCQ has also anti-thrombotic properties via multiple pathways, purportedly through reduced red cell sludging and blood viscosity and possibly through some inhibition of platelet reactivity. In patients with SLE and persistently positive LA, primary thromboprophylaxis with hydroxychloroquine and low-dose aspirin is recommended [71]. The efficacy of HCQ in primary prophylaxis was addressed in an RCT including 22 aPL-positive patients without other autoimmune diseases. No thrombotic events have been reported neither in the LDA arm nor in LDA plus HCQ after a mean follow-up of 1.7 years [72]. In a more recent randomized open-label prospective trial, primary APS patients receiving HCQ, as an adjuvant therapy to standard of care, experienced significantly lower thrombotic rates than those treated with standard of care alone (1/25 vs. 6/25, log-rank *p* = 0.048) over an average 2.6-year follow-up. Moreover, HCQ was associated with downtrending aPL titers [73].

#### 5.4.2. Statins

Statins exert many pleiotropic effects, namely anti-inflammatory and anti-thrombotic, in addition to their action as lipid-lowering drugs. Although some studies showed some promising preliminary results in thrombotic APS patients, the clinical efficacy of statins in this group of patients has not yet been tested in RCTs [74]. In an open-label pilot study, fluvastatin 80 mg daily was given for three months to 41 APS patients. Proinflammatory and prothrombotic biomarkers were significantly reduced by fluvastatin administration. During the study period, only one patient developed DVT. However, it should be taken into account that this study was not designed to assess clinical outcomes [74]. 

In the setting of obstetric APS, pravastatin 20 mg daily was given to APS patients with a history of preeclampsia or intrauterine growth restriction despite treatment with LDA and LMWH. Pravastatin given in addition to LDA and LMWH improved significantly placental blood flow and maternal signs of preeclampsia than standard of care alone [75].

Statins were recommended by the 15th international congress on aPL for APS patients with a high cardiovascular risk and those with recurrent thrombosis despite adequate anticoagulation [71].

#### 5.4.3. Drugs Targeting B-Cell

In a small pilot open-label phase II trial including 20 patients with primary APS, rituximab, a chimeric monoclonal antibody targeting CD20-positive cells, yielded a potential clinical effectiveness in microangiopathic manifestations, such as skin ulcers and aPL nephropathy [76]. The rationale of its administration in APS patients derived from the key role exerted by B cells in APS pathogenesis [77].

#### 5.4.4. Complement Inhibitors

As outlined earlier, complement activation plays a critical role in aPL-induced thrombosis and activates neutrophils to release procoagulant substances [78]. Therefore, treatments targeting the complement pathways might be an attractive alternative for refractory APS patients. Eculizumab, a humanized monoclonal antibody that binds to the C5 protein, has been used as rescue therapy in refractory cases of CAPS as well as in APS patients undergoing renal transplantation [79,80].

#### 5.4.5. Others

In case of immune-mediated thrombocytopenia and haemolytic anaemia, patients may benefit from glucocorticoids with or without intravenous immunoglobulins (IVIGs). Second-line therapies include azathioprine, mycophenolate mofetil, and cyclophosphamide [31].

### 5.5. CAPS

Although many advances in treatment have been made, the mortality rates associated with CAPS remain as high as 48% [27]. Treatment should be carried out by an interdisciplinary team often with access to intensive care unit. The management of CAPS must be initiated as soon as possible and include a combination of anticoagulants, high-dose glucocorticoids, and plasma exchange and/or IVIG, as well as the treatment of any triggering factor [29]. The 15th International Congress on aPL Task Force on Treatment Trends and EULAR concluded that Eculizumab may have a role as an adjuvant therapy for patients refractory to previous treatment [29,71]. It should be kept in mind that treatment of patients with CAPS is based solely on low-quality evidence.

## 6. Conclusions

Many factors are known to influence clinical manifestations, treatment strategies, and the prognosis of patients with APS. The most relevant are aPL profile (aPL type, number, and serum level), the type of the involved vessel, and other cardiovascular risk factors.

APS patients with thrombotic events should benefit from a tight control of major thrombosis risk factors, such as oestrogen, major surgeries, long flights, and immobilization as well as traditional cardiovascular risk factors.

APS patients with thromboembolism should be treated with long-term VKA at a target INR of 2–3; for those presenting with arterial events, either high-intensity anticoagulation or combined therapy with LDA and standard-intensity warfarin are the best strategies.

It is recommended to avoid DOACs in patients with high-risk APS, mainly those with triple-positive PL and previous arterial events. They would only be used exceptionally in selected patients with low-risk VTE. Results of ongoing clinical trials will indicate whether it is safe to continue DOACs in the specific context of low-risk APS patients, and which DOAC should be considered. In women with obstetric APS, the combination therapy with LDA plus heparin remains the conventional strategy.

The management of APS should be patient centred, taking into account risk stratification, clinical phenotype, and comorbidities. Patients with APS should benefit from a tight control to monitor the efficacy and the safety of anti-thrombotic therapy. 

## Figures and Tables

**Table 1:** When to order antiphospholipid antibodies (aPL) testing, Adapted from [16].

▪ History of systemic lupus erythematosus (SLE) or other autoimmune disease
▪ Presence of livedo reticularis
▪ Prolonged activated partial thromboplastin time (APTT) prior to starting anticoagulation
▪ Recurrent thrombosis
▪ VTE at unusual sites
▪ History of arterial thrombosis without clear risk factors
▪ Thrombocytopenia
▪ Recurrent miscarriages/still birth/severe pre-eclampsia
▪ Cardiac valve abnormalities in the absence of other explanations

Adapted from [17].

**Table 2:** Clinical trials of direct oral anticoagulants for thrombotic antiphospholipid syndrome.

	RAPS [50]	Re-Cover, Re-Cover II, and Remedy (Post-Hoc) [81]	TRAPS [51]	EUDRA [53]
Publication year	2016	2016	2018	2019
Study design	Open label, phase II/III, non-inferiority RCT	Double-dummy, non-inferiority RCT	Open label, phase III, non-inferiority RCT	Open label, non-inferiority RCT
Sample size (DOAC/VKA)	116 (57/59)	151 (71/80)	120 (59/61)	190 (95/95)
% Triple positivity (DOACs/VKA)	12/20	NA	100/100	61.1/60
% prior arterial APS (DOACs/VKA)	0/0	NA	19/23	38.9/35.8
DOAC/dose (mg)	Rivaroxaban/20 OD	Dabigatran/150 BID	Rivaroxaban/20 OD	Rivaroxaban/20 OD
VKA/target INR	Warfarin/2.5	Warfarin/2.5	Warfarin/2.5	Warfarin/2.5
Follow-up (months)	7	36	8	36
% Thrombosis (DOAC/VKA), HR 95%CI	0/0, not applicable	4.2/5.0 0.43 (0.08–2.38) ^1^	12.0/0 7.4 (1.7–32.9) ^2^	11.6/6.3 1.94 (0.72–5.24)
% Major bleeding (DOAC/VKA), HR 95%CI	0/0, not applicable	1.4/2.6 0.46 (0.04–5.43)	7.0/3.0 7.4 (1.7–32.9)	9.5/5.3 0.88(0.30–2.63)

^1^: Hazard ratio for Composite outcome, including venous thromboembolism and venous thromboembolism-related death. ^2^: Hazard ratio for composite outcome, including thromboembolic events, major bleeding, and vascular death. APS = antiphospholipid syndrome; BID = twice daily; CI = confidence interval; DOAC = direct oral anticoagulant; HR = hazard ratio; INR = international normalized ratio; NA = not available; OD = once daily; RCT = randomized controlled trial; VKA = vitamin k antagonist.

## References

[1] Giannakopoulos B., Passam F., Ioannou Y., Krilis S.A. (2009). How we diagnose the antiphospholipid syndrome. Blood.

[2] Jayakody Arachchillage D., Greaves M. (2014). The chequered history of the antiphospholipid syndrome. Br. J. Haematol..

[3] Mezhov V., Segan J.D., Tran H., Cicuttini F.M. (2019). Antiphospholipid syndrome: A clinical review. Med. J. Aust..

[4] Tektonidou M.G., Laskari K., Panagiotakos D.B., Moutsopoulos H.M. (2009). Risk factors for thrombosis and primary thrombosis prevention in patients with systemic lupus erythematosus with or without antiphospholipid antibodies. Arthritis Care Res..

[5] Cervera R., Boffa M.-C., Khamashta M.A., Hughes G.R.V. (2009). The Euro-Phospholipid project: Epidemiology of the antiphospholipid syndrome in Europe. Lupus.

[6] Alijotas-Reig J., Esteve-Valverde E., Ferrer-Oliveras R., LLurba E., Ruffatti A., Tincani A., Lefkou E., Bertero M.T., Espinosa G., de Carolis S. (2018). Comparative study between obstetric antiphospholipid syndrome and obstetric morbidity related with antiphospholipid antibodies. Med. Clin..

[7] de Jesús G.R., Benson A.E., Chighizola C.B., Sciascia S., Branch D.W. (2020). Sixteenth international congress on antiphospholipid antibodies task force. Report on obstetric antiphospholipid syndrome. Lupus.

[8] Meroni P.L., Borghi M.O., Grossi C., Chighizola C.B., Durigutto P., Tedesco F. (2018). Obstetric and vascular antiphospholipid syndrome: Same antibodies but different diseases?. Nat. Rev. Rheumatol..

[9] de Jesús G.R., Sciascia S., Andrade D., Barbhaiya M., Tektonidou M., Banzato A., Pengo V., Ji L., Meroni P.L., Ugarte A. (2019). Factors associated with first thrombosis in patients presenting with obstetric antiphospholipid syndrome (APS) in the APS Alliance for Clinical Trials and International Networking Clinical Database and Repository: A retrospective study. BJOG Int. J. Obstet. Gynaecol..

[10] Sciascia S., Sanna G., Murru V., Roccatello D., Khamashta M.A., Bertolaccini M.L. (2015). The global anti-phospholipid syndrome score in primary APS. Rheumatol. Oxf. Engl..

[11] Meroni P.L., Chighizola C.B., Rovelli F., Gerosa M. (2014). Antiphospholipid syndrome in 2014: More clinical manifestations, novel pathogenic players and emerging biomarkers. Arthritis Res. Ther..

[12] Amengual O., Fujita D., Ota E., Carmona L., Oku K., Sugiura-Ogasawara M., Murashima A., Atsumi T. (2015). Primary prophylaxis to prevent obstetric complications in asymptomatic women with antiphospholipid antibodies: A systematic review. Lupus.

[13] Cohen H., Efthymiou M., Isenberg D.A. (2018). Use of direct oral anticoagulants in antiphospholipid syndrome. J. Thromb. Haemost..

[14] Duarte-García A., Pham M.M., Crowson C.S., Amin S., Moder K.G., Pruthi R.K., Warrington K.J., Matteson E.L. (2019). The Epidemiology of Antiphospholipid Syndrome: A Population-Based Study. Arthritis Rheumatol..

[15] Dafer R.M., Biller J. (2008). Antiphospholipid syndrome: Role of antiphospholipid antibodies in neurology. Hematol. Oncol. Clin. N. Am..

[16] Ruffatti A., Del Ross T., Ciprian M., Bertero M.T., Sciascia S., Salvatore S., Scarpato S., Montecucco C., Rossi S., Caramaschi P. (2011). Risk factors for a first thrombotic event in antiphospholipid antibody carriers: A prospective multicentre follow-up study. Ann. Rheum. Dis..

[17] Arachchillage D.R.J., Gomez K., Alikhan R., Anderson J.A.M., Lester W., Laffan M., British Society for Haematology Haemostasis and Thrombosis Taskforce Addendum to British Society for Haematology Guidelines on Investigation and Management of Antiphospholipid syndrome, 2012 (Br (2020). J. Haematol. 2012; 157: 47-58): Use of direct acting oral anticoagulants. Br. J. Haematol..

[18] Chighizola C.B., Sciascia S., Andreoli L., Meroni P.L. (2020). Beyond current concepts in anti-phospholipid syndrome: The 16th International Congress on Anti-phospholipid Antibodies (ICAPA) in Manchester. Autoimmun. Rev..

[19] Nakamura H., Oku K., Amengual O., Ohmura K., Fujieda Y., Kato M., Bohgaki T., Yasuda S., Atsumi T. (2018). First-Line, Non-Criterial Antiphospholipid Antibody Testing for the Diagnosis of Antiphospholipid Syndrome in Clinical Practice: A Combination of Anti–β2-Glycoprotein I Domain I and Anti–Phosphatidylserine/Prothrombin Complex Antibodies Tests. Arthritis Care Res..

[20] Hughes G.R.V., Khamashta M.A. (2003). Seronegative antiphospholipid syndrome. Ann. Rheum. Dis..

[21] Zohoury N., Bertolaccini M.L., Rodriguez-Garcia J.L., Shums Z., Ateka-Barrutia O., Sorice M., Norman G.L., Khamashta M. (2017). Closing the Serological Gap in the Antiphospholipid Syndrome: The Value of “Non-criteria” Antiphospholipid Antibodies. J. Rheumatol..

[22] Bertolaccini M.L., Amengual O., Atsumi T., Binder W.L., de Laat B., Forastiero R., Kutteh W.H., Lambert M., Matsubayashi H., Murthy V. (2011). ‘Non-criteria’ aPL tests: Report of a task force and preconference workshop at the 13th International Congress on Antiphospholipid Antibodies, Galveston, TX, USA, April 2010. Lupus.

[23] Miyakis S., Lockshin M.D., Atsumi T., Branch D.W., Brey R.L., Cervera R., Derksen R.H.W.M., DE Groot P.G., Koike T., Meroni P.L. (2006). International consensus statement on an update of the classification criteria for definite antiphospholipid syndrome (APS). J. Thromb. Haemost..

[24] Devignes J., Smaïl-Tabbone M., Hervé A., Cagninacci G., Devignes M.-D., Lecompte T., Zuily S., Wahl D. (2019). Extended persistence of antiphospholipid antibodies beyond the 12-week time interval: Association with baseline antiphospholipid antibodies titres. Int. J. Lab. Hematol..

[25] Rodríguez-Pintó I., Espinosa G., Erkan D., Shoenfeld Y., Cervera R. (2018). CAPS Registry Project Group The effect of triple therapy on the mortality of catastrophic anti-phospholipid syndrome patients. Rheumatol. Oxf. Engl..

[26] Asherson R.A., Cervera R., de Groot P.G., Erkan D., Boffa M.-C., Piette J.-C., Khamashta M.A., Shoenfeld Y., Group C.A.S.R.P. (2016). Catastrophic antiphospholipid syndrome: International consensus statement on classification criteria and treatment guidelines. Lupus.

[27] Rodríguez-Pintó I., Moitinho M., Santacreu I., Shoenfeld Y., Erkan D., Espinosa G., Cervera R. (2016). Catastrophic antiphospholipid syndrome (CAPS): Descriptive analysis of 500 patients from the International CAPS Registry. Autoimmun. Rev..

[28] Asherson R.A., Cervera R. (2003). Catastrophic antiphospholipid syndrome. Curr. Rheumatol. Rep..

[29] Tektonidou M.G., Andreoli L., Limper M., Amoura Z., Cervera R., Costedoat-Chalumeau N., Cuadrado M.J., Dörner T., Ferrer-Oliveras R., Hambly K. (2019). EULAR recommendations for the management of antiphospholipid syndrome in adults. Ann. Rheum. Dis..

[30] Sciascia S., Sanna G., Murru V., Roccatello D., Khamashta M.A., Bertolaccini M.L. (2013). GAPSS: The Global Anti-Phospholipid Syndrome Score. Rheumatology.

[31] Garcia D., Erkan D. (2018). Diagnosis and Management of the Antiphospholipid Syndrome. N. Engl. J. Med..

[32] Erkan D., Harrison M.J., Levy R., Peterson M., Petri M., Sammaritano L., Unalp-Arida A., Vilela V., Yazici Y., Lockshin M.D. (2007). Aspirin for primary thrombosis prevention in the antiphospholipid syndrome: A randomized, double-blind, placebo-controlled trial in asymptomatic antiphospholipid antibody-positive individuals. Arthritis Rheum..

[33] Arnaud L., Mathian A., Ruffatti A., Erkan D., Tektonidou M., Cervera R., Forastiero R., Pengo V., Lambert M., Martinez-Zamora M.A. (2014). Efficacy of aspirin for the primary prevention of thrombosis in patients with antiphospholipid antibodies: An international and collaborative meta-analysis. Autoimmun. Rev..

[34] Arnaud L., Mathian A., Devilliers H., Ruffatti A., Tektonidou M., Forastiero R., Pengo V., Lambert M., Lefevre G., Martinez-Zamora M.A. (2015). Patient-level analysis of five international cohorts further confirms the efficacy of aspirin for the primary prevention of thrombosis in patients with antiphospholipid antibodies. Autoimmun. Rev..

[35] Collaboration A.T. (2009). (ATT) Aspirin in the primary and secondary prevention of vascular disease: Collaborative meta-analysis of individual participant data from randomised trials. Lancet.

[36] Wahl D.G., Bounameaux H., de Moerloose P., Sarasin F.P. (2000). Prophylactic antithrombotic therapy for patients with systemic lupus erythematosus with or without antiphospholipid antibodies: Do the benefits outweigh the risks? A decision analysis. Arch. Intern. Med..

[37] Cuadrado M.J., Bertolaccini M.L., Seed P.T., Tektonidou M.G., Aguirre A., Mico L., Gordon C., Ruiz-Irastorza G., Egurbide M.V., Gil A. (2014). Low-dose aspirin vs low-dose aspirin plus low-intensity warfarin in thromboprophylaxis: A prospective, multicentre, randomized, open, controlled trial in patients positive for antiphospholipid antibodies (ALIWAPAS). Rheumatol. Oxf. Engl..

[38] Ruiz-Irastorza G., Cuadrado M.J., Ruiz-Arruza I., Brey R., Crowther M., Derksen R., Erkan D., Krilis S., Machin S., Pengo V. (2011). Evidence-based recommendations for the prevention and long-term management of thrombosis in antiphospholipid antibody-positive patients: Report of a task force at the 13th International Congress on antiphospholipid antibodies. Lupus.

[39] Arnaud L., Conti F., Massaro L., Denas G., Chasset F., Pengo V. (2017). Primary thromboprophylaxis with low-dose aspirin and antiphospholipid antibodies: Pro’s and Con’s. Autoimmun. Rev..

[40] Lim W., Crowther M.A., Eikelboom J.W. (2006). Management of antiphospholipid antibody syndrome: A systematic review. JAMA.

[41] Rosove M.H., Brewer P.M. (1992). Antiphospholipid thrombosis: Clinical course after the first thrombotic event in 70 patients. Ann. Intern. Med..

[42] Crowther M.A., Ginsberg J.S., Julian J., Denburg J., Hirsh J., Douketis J., Laskin C., Fortin P., Anderson D., Kearon C. (2003). A comparison of two intensities of warfarin for the prevention of recurrent thrombosis in patients with the antiphospholipid antibody syndrome. N. Engl. J. Med..

[43] Finazzi G., Marchioli R., Brancaccio V., Schinco P., Wisloff F., Musial J., Baudo F., Berrettini M., Testa S., D’Angelo A. (2005). A randomized clinical trial of high-intensity warfarin vs. conventional antithrombotic therapy for the prevention of recurrent thrombosis in patients with the antiphospholipid syndrome (WAPS). J. Thromb. Haemost..

[44] Levine S.R., Brey R.L., Tilley B.C., Thompson J.L.P., Sacco R.L., Sciacca R.R., Murphy A., Lu Y., Costigan T.M., Rhine C. (2004). Antiphospholipid antibodies and subsequent thrombo-occlusive events in patients with ischemic stroke. JAMA.

[45] Okuma H., Kitagawa Y., Yasuda T., Tokuoka K., Takagi S. (2009). Comparison between single antiplatelet therapy and combination of antiplatelet and anticoagulation therapy for secondary prevention in ischemic stroke patients with antiphospholipid syndrome. Int. J. Med. Sci..

[46] Cohen H., Isenberg D. (2020). How I treat anticoagulant-refractory thrombotic antiphospholipid syndrome. Blood.

[47] Wiggins B.S., Dixon D.L., Neyens R.R., Page R.L., Gluckman T.J. (2020). Select Drug-Drug Interactions with Direct Oral Anticoagulants: JACC Review Topic of the Week. J. Am. Coll. Cardiol..

[48] Chen A., Stecker E., Warden B.A. (2020). Direct Oral Anticoagulant Use: A Practical Guide to Common Clinical Challenges. J. Am. Heart Assoc..

[49] Dufrost V., Risse J., Reshetnyak T., Satybaldyeva M., Du Y., Yan X.-X., Salta S., Gerotziafas G., Jing Z.-C., Elalamy I. (2018). Increased risk of thrombosis in antiphospholipid syndrome patients treated with direct oral anticoagulants. Results from an international patient-level data meta-analysis. Autoimmun. Rev..

[50] Cohen H., Hunt B.J., Efthymiou M., Arachchillage D.R.J., Mackie I.J., Clawson S., Sylvestre Y., Machin S.J., Bertolaccini M.L., Ruiz-Castellano M. (2016). Rivaroxaban versus warfarin to treat patients with thrombotic antiphospholipid syndrome, with or without systemic lupus erythematosus (RAPS): A randomised, controlled, open-label, phase 2/3, non-inferiority trial. Lancet Haematol..

[51] Pengo V., Denas G., Zoppellaro G., Jose S.P., Hoxha A., Ruffatti A., Andreoli L., Tincani A., Cenci C., Prisco D. (2018). Rivaroxaban vs warfarin in high-risk patients with antiphospholipid syndrome. Blood.

[52] Sanchez-Redondo J., Espinosa G., Varillas Delgado D., Cervera R. (2019). Recurrent Thrombosis with Direct Oral Anticoagulants in Antiphospholipid Syndrome: A Systematic Literature Review and Meta-analysis. Clin. Ther..

[53] Ordi-Ros J., Sáez-Comet L., Pérez-Conesa M., Vidal X., Riera-Mestre A., Castro-Salomó A., Cuquet-Pedragosa J., Ortiz-Santamaria V., Mauri-Plana M., Solé C. (2019). Rivaroxaban Versus Vitamin K Antagonist in Antiphospholipid Syndrome. Ann. Intern. Med..

[54] PRAC Recommendations on Signals. https://www.ema.europa.eu/en/human-regulatory/post-authorisation/pharmacovigilance/signal-management/prac-recommendations-safety-signals.

[55] Sciascia S., Coloma-Bazán E., Radin M., Bertolaccini M.L., López-Pedrera C., Espinosa G., Meroni P.L., Cervera R., Cuadrado M.J. (2017). Can we withdraw anticoagulation in patients with antiphospholipid syndrome after seroconvertion?. Autoimmun. Rev..

[56] Andreoli L., Bertsias G.K., Agmon-Levin N., Brown S., Cervera R., Costedoat-Chalumeau N., Doria A., Fischer-Betz R., Forger F., Moraes-Fontes M.F. (2017). EULAR recommendations for women’s health and the management of family planning, assisted reproduction, pregnancy and menopause in patients with systemic lupus erythematosus and/or antiphospholipid syndrome. Ann. Rheum. Dis..

[57] Massimo R., Irene C., Elena R., Grazietta F.S., Alice B., Elisa M., Dario R., Sciascia S. (2020). Treatment of antiphospholipid syndrome. Clin. Immunol..

[58] Kahwa E.K., Sargeant L.A., McCaw-Binns A., McFarlane-Anderson N., Smikle M., Forrester T., Wilks R. (2006). Anticardiolipin antibodies in Jamaican primiparae. J. Obstet. Gynaecol..

[59] Liu X., Qiu Y., Yu E.D., Xiang S., Meng R., Niu K.F., Zhu H. (2020). Comparison of therapeutic interventions for recurrent pregnancy loss in association with antiphospholipid syndrome: A systematic review and network meta-analysis. Am. J. Reprod. Immunol..

[60] Jeremic K., Stefanovic A., Dotlic J., Stojnic J., Kadija S., Vilendecic Z., Janjic T., Jeremic J. (2015). Neonatal outcome in pregnant patients with antiphospholipid syndrome. J. Perinat. Med..

[61] Lu C., Liu Y., Jiang H.-L. (2019). Aspirin or heparin or both in the treatment of recurrent spontaneous abortion in women with antiphospholipid antibody syndrome: A meta-analysis of randomized controlled trials. J. Matern. Fetal Neonatal Med..

[62] Lockshin M.D., Kim M., Laskin C.A., Guerra M., Branch D.W., Merrill J., Petri M., Porter T.F., Sammaritano L., Stephenson M.D. (2012). Prediction of adverse pregnancy outcome by the presence of lupus anticoagulant, but not anticardiolipin antibody, in patients with antiphospholipid antibodies. Arthritis Rheum..

[63] Mekinian A., Lazzaroni M.G., Kuzenko A., Alijotas-Reig J., Ruffatti A., Levy P., Canti V., Bremme K., Bezanahary H., Bertero T. (2015). The efficacy of hydroxychloroquine for obstetrical outcome in anti-phospholipid syndrome: Data from a European multicenter retrospective study. Autoimmun. Rev..

[64] Sciascia S., Hunt B.J., Talavera-Garcia E., Lliso G., Khamashta M.A., Cuadrado M.J. (2016). The impact of hydroxychloroquine treatment on pregnancy outcome in women with antiphospholipid antibodies. Am. J. Obstet. Gynecol..

[65] Schreiber K., Breen K., Cohen H., Jacobsen S., Middeldorp S., Pavord S., Regan L., Roccatello D., Robinson S.E., Sciascia S. (2017). HYdroxychloroquine to Improve Pregnancy Outcome in Women with AnTIphospholipid Antibodies (HYPATIA) Protocol: A Multinational Randomized Controlled Trial of Hydroxychloroquine versus Placebo in Addition to Standard Treatment in Pregnant Women with Antiphospholipid Syndrome or Antibodies. Semin. Thromb. Hemost..

[66] Mekinian A., Vicaut E., Cohen J., Bornes M., Kayem G., Fain O. (2018). [Hydroxychloroquine to obtain pregnancy without adverse obstetrical events in primary antiphospholipid syndrome: French phase II multicenter randomized trial, HYDROSAPL]. Gynecol. Obstet. Fertil. Senol..

[67] Belizna C., Pregnolato F., Abad S., Alijotas-Reig J., Amital H., Amoura Z., Andreoli L., Andres E., Aouba A., Apras Bilgen S. (2018). HIBISCUS: Hydroxychloroquine for the secondary prevention of thrombotic and obstetrical events in primary antiphospholipid syndrome. Autoimmun. Rev..

[68] Pignatelli P., Ettorre E., Menichelli D., Pani A., Violi F., Pastori D. (2020). Seronegative antiphospholipid syndrome: Refining the value of “non-criteria” antibodies for diagnosis and clinical management. Haematologica.

[69] Mekinian A., Bourrienne M.-C., Carbillon L., Benbara A., Noémie A., Chollet-Martin S., Tigaizin A., Montestruc F., Fain O., Nicaise-Roland P. (2016). Non-conventional antiphospholipid antibodies in patients with clinical obstetrical APS: Prevalence and treatment efficacy in pregnancies. Semin. Arthritis Rheum..

[70] Abisror N., Nguyen Y., Marozio L., Valverde E.E., Udry S., Pleguezuelo D.E., Billoir P., Mayer-Pickel K., Urbanski G., Zigon P. (2020). Obstetrical outcome and treatments in seronegative primary APS: Data from European retrospective study. RMD Open.

[71] Andrade D., Cervera R., Cohen H., Crowther M., Cuadrado M.J., Canaud G., Garcia D.A., Gerosa M., Ortel T.L., Pengo V., Erkan D., Lockshin M.D. (2017). 15th International Congress on Antiphospholipid Antibodies Task Force on Antiphospholipid Syndrome Treatment Trends Report. Antiphospholipid Syndrome: Current Research Highlights and Clinical Insights.

[72] Erkan D., Unlu O., Sciascia S., Belmont H.M., Branch D.W., Cuadrado M.J., Gonzalez E., Knight J.S., Uthman I., Willis R. (2017). Hydroxychloroquine in the primary thrombosis prophylaxis of antiphospholipid antibody positive patients without systemic autoimmune disease. Lupus.

[73] Kravvariti E., Koutsogianni A., Samoli E., Sfikakis P.P., Tektonidou M.G. (2020). The effect of hydroxychloroquine on thrombosis prevention and antiphospholipid antibody levels in primary antiphospholipid syndrome: A pilot open label randomized prospective study. Autoimmun. Rev..

[74] Erkan D., Willis R., Murthy V.L., Basra G., Vega J., Ruiz-Limón P., Carrera A.L., Papalardo E., Martínez-Martínez L.A., González E.B. (2014). A prospective open-label pilot study of fluvastatin on proinflammatory and prothrombotic biomarkers in antiphospholipid antibody positive patients. Ann. Rheum. Dis..

[75] Lefkou E., Mamopoulos A., Dagklis T., Vosnakis C., Rousso D., Girardi G. (2016). Pravastatin improves pregnancy outcomes in obstetric antiphospholipid syndrome refractory to antithrombotic therapy. J. Clin. Investig..

[76] Erkan D., Vega J., Ramón G., Kozora E., Lockshin M.D. (2013). A pilot open-label phase II trial of rituximab for non-criteria manifestations of antiphospholipid syndrome. Arthritis Rheum..

[77] Khattri S., Zandman-Goddard G., Peeva E. (2012). B-cell directed therapies in antiphospholipid antibody syndrome--new directions based on murine and human data. Autoimmun. Rev..

[78] Pierangeli S.S., Girardi G., Vega-Ostertag M., Liu X., Espinola R.G., Salmon J. (2005). Requirement of activation of complement C3 and C5 for antiphospholipid antibody–mediated thrombophilia. Arthritis Rheum..

[79] Lonze B.E., Zachary A.A., Magro C.M., Desai N.M., Orandi B.J., Dagher N.N., Singer A.L., Carter-Monroe N., Nazarian S.M., Segev D.L. (2014). Eculizumab prevents recurrent antiphospholipid antibody syndrome and enables successful renal transplantation. Am. J. Transplant. Off. J. Am. Soc. Transplant. Am. Soc. Transpl. Surg..

[80] Yelnik C.M., Miranda S., Mékinian A., Lazaro E., Quéméneur T., Provot F., Frimat M., Morell-Dubois S., Le Guern V., Hachulla E. (2020). Refractory Catastrophic Antiphospholipid Syndrome patients respond inconsistently to Eculizumab. Blood.

[81] Goldhaber S.Z., Eriksson H., Kakkar A., Schellong S., Feuring M., Fraessdorf M., Kreuzer J., Schueler E., Schulman S. (2016). Efficacy of dabigatran versus warfarin in patients with acute venous thromboembolism in the presence of thrombophilia: Findings from RE-COVER^®^, RE-COVER^TM^ II, and RE-MEDY^TM^. Vasc. Med..

